# Fitness estimates from experimental infections predict the long-term strain structure of a vector-borne pathogen in the field

**DOI:** 10.1038/s41598-017-01821-1

**Published:** 2017-05-12

**Authors:** Jonas Durand, Maxime Jacquet, Olivier Rais, Lise Gern, Maarten J. Voordouw

**Affiliations:** 1 Laboratory of Ecology and Evolution of Parasites, Institute of Biology, University of Neuchâtel, Neuchâtel, Switzerland; 2 Laboratory of Eco-Epidemiology of Parasites, Institute of Biology, University of Neuchâtel, Neuchâtel, Switzerland

## Abstract

The populations of many pathogen species consist of a collection of common and rare strains but the factors underlying this strain-specific variation in frequency are often unknown. Understanding frequency variation among strains is particularly challenging for vector-borne pathogens where the strain-specific fitness depends on the performance in both the vertebrate host and the arthropod vector. Two sympatric multiple-strain tick-borne pathogens, *Borrelia afzelii* and *B. garinii*, that use the same tick vector, *Ixodes ricinus*, but different vertebrate hosts were studied. 454-sequencing of the polymorphic *ospC* gene was used to characterize the community of *Borrelia* strains in a local population of *I. ricinus* ticks over a period of 11 years. Estimates of the reproduction number (R_0_), a measure of fitness, were obtained for six strains of *B. afzelii* from a previous laboratory study. There was substantial variation in prevalence among strains and some strains were consistently common whereas other strains were consistently rare. In *B. afzelii*, the strain-specific estimates of R_0_ in laboratory mice explained over 70% of the variation in the prevalences of the strains in our local population of ticks. Our study shows that laboratory estimates of fitness can predict the community structure of multiple-strain pathogens in the field.

## Introduction

Many pathogen populations consist of multiple strains or mixed infections in their host^1–3^. From a public health perspective, the study of multiple-strain pathogens and parasites is important for a number of reasons. Strains belonging to the same pathogen species can show enormous variation in their ability to establish infection and cause disease^4, 5^. Mixed infections can induce more pathology and disease than single-strain infections^6–8^. Multiple-strain pathogens complicate the development of vaccines and anti-parasite drugs^2, 9^. The use of vaccines and anti-parasite drugs can induce strong selection on multiple-strain pathogen populations and result in the competitive release of strains that are not targeted by human medicine^10–13^. Multiple-strain infections are of further interest to evolutionary biologists because competition between strains influences the evolution of virulence^3^.

Studies on multiple-strain pathogens have shown that some strains are consistently more common than others over time and/or space^14–17^. A fundamental question is therefore to understand the genetic and phenotypic factors that underlie this variation in frequency among strains. For example, in *Streptococcus pneumonia*, the thickness and structure of the capsule were good predictors of whether a given serotype was common or not^16^. In the case of vector-borne pathogens, the search for predictive phenotypes is complicated by the fact that the frequency of a particular strain depends on its performance in both the vertebrate host and the arthropod vector. Recent developments in so-called next generation population matrix models now allow scientists to combine the relevant transmission components of vector-borne pathogens into the reproduction number (R_0_), an inclusive measure of fitness^18, 19^. In conjunction, the development of next generation sequencing methods has greatly enhanced our ability to detect multiple infections in individual hosts^20, 21^. In the present study, we combined these two next generation methods to better understand the strain-specific prevalence distribution in a tick-borne pathogen.

*Borrelia afzelii* and *B. garinii* are two species of tick-borne spirochete bacteria that cause Lyme borreliosis in humans^22^. In Europe, the main vector for both pathogen species is the hard tick *Ixodes ricinus*. The two immature tick stages, larva and nymph, take a single blood meal to develop into the next stage^23^. Larval ticks acquire spirochetes after feeding on an infected reservoir host and develop into infected nymphs that transmit the pathogen the following year to the next generation of reservoir hosts. *B. afzelii* and *B. garinii* are specialized on different classes of vertebrate hosts: rodents and birds, respectively^23–26^, and therefore rarely occur together in the same tick^27–30^. Local populations of both *Borrelia* species consist of multiple strains^21, 31–34^, which are often defined by the highly polymorphic, single-copy *ospC* gene^35–38^. In the present study, we used the *ospC*-typing system to study mixed-strain infections within each of the two *Borrelia* species, as others have done previously^21, 31, 33, 34, 39–42^.

The first purpose of the present study was to test whether laboratory estimates of strain-specific fitness could predict the strain structure of *Borrelia* pathogens in the field. We had recently used a laboratory Lyme borreliosis system that included *Mus musculus* mice and *I. ricinus* ticks to show that there was significant variation in fitness among six different *ospC* strains of *B. afzelii* ^43^. For each of the six strains, we measured the three most important fitness components of any vector-borne pathogen: vector-to-host transmission, host-to-vector transmission, and co-feeding transmission^43^. We then used next generation matrices to estimate the reproduction number (R_0_) for each of the six strains^43^. These strains originated from a field site near Neuchâtel where the local tick population had been sampled over a period of 11 years to create a collection of isolates of *B. afzelii* and *B. garinii*. We had recently estimated the diversity of *ospC* strains in these tick-derived isolates using 454-sequencing^21^. These two studies therefore provided a unique opportunity to test whether laboratory estimates of strain-specific fitness can predict the strain structure of *Borrelia* pathogens in the field.

The second purpose of the present study was to test whether the prevalences of the different *ospC* strains were stable or fluctuating over time. A review on the evolutionary ecology of LB pathogens emphasized the importance of studying temporal variation in the frequencies of *Borrelia ospC* strains^22^. A number of Lyme disease researchers have predicted that the frequencies of the *ospC* strains should cycle over time^22, 44, 45^. Thus long-term studies are important because they may improve our understanding of the ecological factors that shape the dynamics of multiple-strain *Borrelia* pathogens. In addition, for the laboratory estimates of fitness to have predictive value, the *ospC* strain structure should be stable over time so that some strains are consistently more common than others. In contrast, if the *ospC* strains cycle between being rare and being common, the laboratory estimates of R_0_ are unlikely to predict the long-term average strain-specific prevalences. The present study therefore provided a unique opportunity to test whether the prevalences of the *ospC* strains are stable or fluctuating over time in two sympatric *Borrelia* species.

## Results

### Background

The University of Neuchâtel has a large collection of *B. afzelii* and *B. garinii* isolates that were obtained from a local population of *I. ricinus* nymphs over a period of 11 years. We had previously characterized the community of *ospC* strains in a stratified random sample of these isolates using 454-sequencing^21^. The stratified random sample contained a maximum of 20 isolates for each of the 22 combinations of *Borrelia* species and year, resulting in a total of 193 isolates of *B. afzelii* and 190 isolates of *B. garinii* ^21^. We had previously assigned the *ospC* gene sequences to a limited set of distinct clusters, the so-called *ospC* major groups (oMGs), of which 10 belonged to *B. afzelii* and 11 to *B. garinii* ^21^. For each oMG strain, the proportion of isolates carrying at least one spirochete of that particular strain was calculated for each year and for the duration of the study. These proportions give the prevalence of each oMG strain in the subset of infected ticks. We use the term ‘relative prevalence’ to indicate that the proportion was calculated over the subset of infected ticks that yielded an isolate of that particular *Borrelia* species and not the whole sample of ticks (i.e., uninfected ticks were excluded in the calculation of this proportion). The prevalence can range from 0.00 to 1.00 for any given oMG strain in any given year.

### Relative prevalences differ among the oMG strains in *B. afzelii* and *B. garinii*

**Figure 1:**
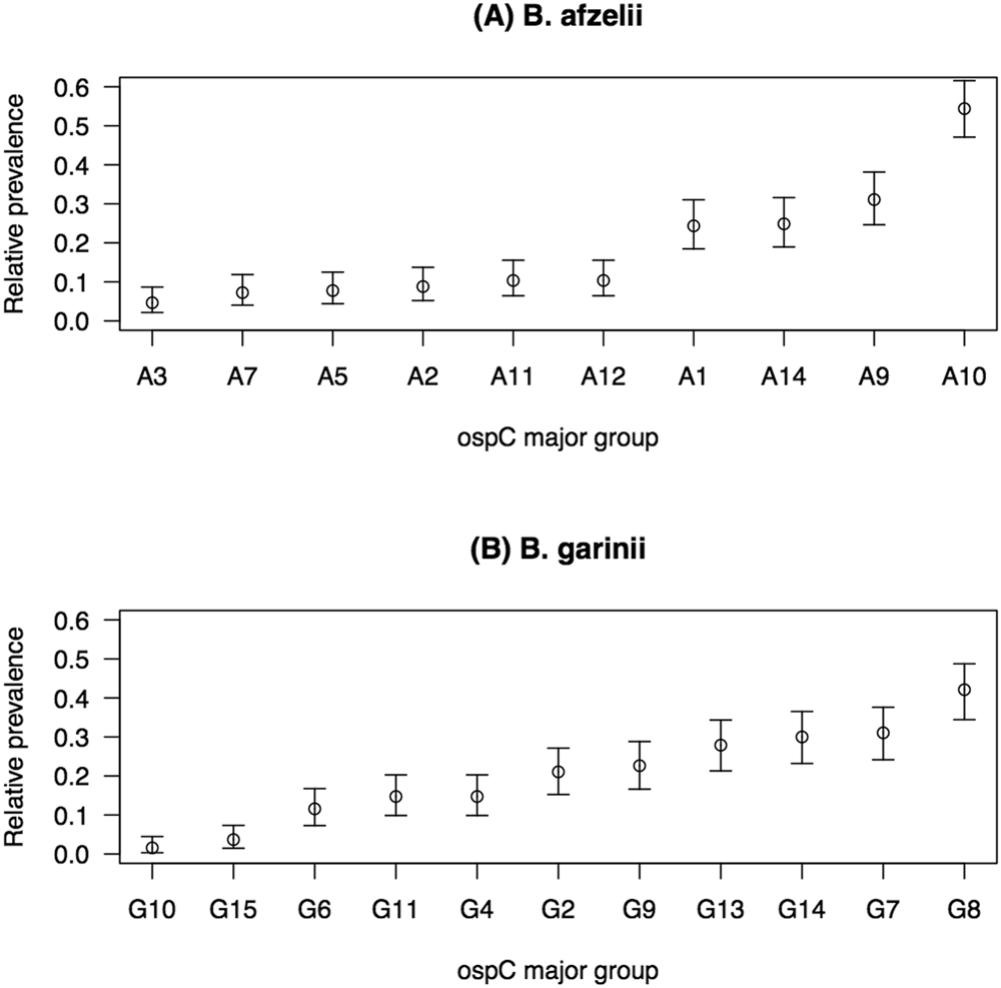
The relative prevalences differ significantly among the oMGs in (**A**) *B. afzelii* and in (**B**) *B. garinii*. For each species, the oMGs are ranked from least common to most common. The relative prevalence of an oMG strain is defined as the proportion of *B. afzelii*-infected nymphs that are infected with that particular oMG strain.

Proportion tests were used to determine whether the long-term average prevalences differed among oMG strains within each *Borrelia* species. The mean relative prevalences were significantly different between the 10 oMG strains in *B. afzelii* (proportion test: χ^2^ = 280.512, df = 9, p < 0.001; Fig. 1) and between the 11 oMG strains in *B. garinii* (proportion test: χ^2^ = 179.018, df = 10, p < 0.001; Fig. 1). In *B. afzelii*, the most common oMG (A10) was 11.7 times more common than the least common oMG (A3). In *B. garinii*, the most common oMG (G8) was 26.7 times more common than the least common oMG (G10).

### Directional change in the relative prevalences of the oMG strains over time

**Figure 2:**
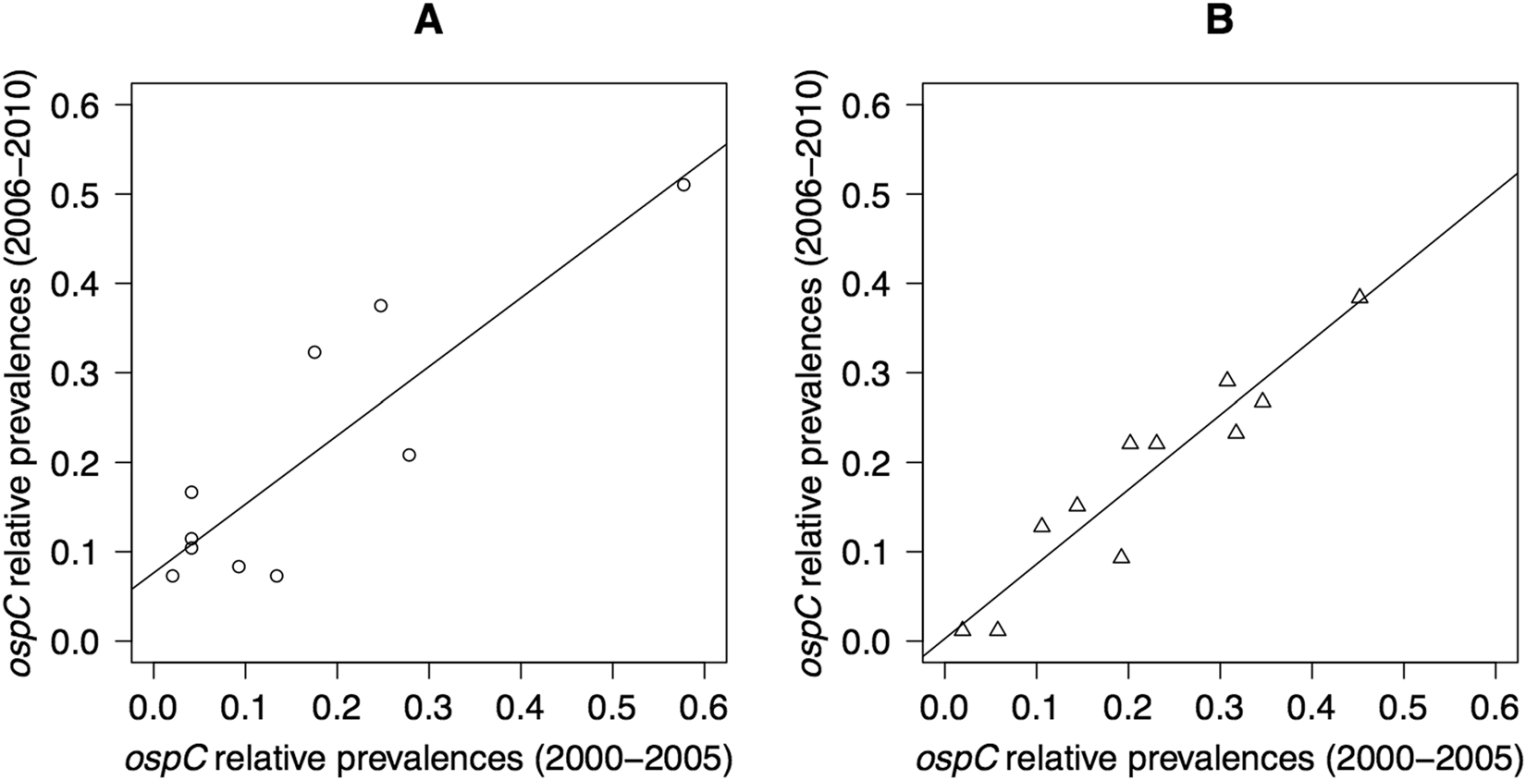
The community of oMG strains is stable over time. The relationship between the relative prevalences of the oMG strains of the first 6 years versus the last 5 years of the survey is shown for both (**A**) *B. afzelii* and (**B**) *B. garinii*. The Pearson correlation of the relative prevalence distribution of the oMG strains between the two time periods was highly significant for both *Borrelia* species. The relative prevalence of an oMG strain is defined as the proportion of *B. afzelii*-infected nymphs that are infected with that particular oMG strain.

The relative prevalence distribution of the oMG strains for the first six years of the study was significantly correlated with the relative prevalence distribution for the last five years of the study in both *B. afzelii* (Pearson correlation test: r = 0.869, p = 0.001; Fig. 2) and *B. garinii* (Pearson correlation test: r = 0.945, p < 0.001; Fig. 2). This result suggests that the community of oMG strains was the same between the two halves of the study, and that previously common strains did not become rare or vice versa. There was significant directional change in the relative prevalence between the start (2000 to 2005) and the end (2006 to 2010) of the study for 2 of the 20 oMGs (Table S2). *B. afzelii* oMGs A11 and A14 increased their relative prevalence by a factor of 4.0 (p = 0.009) and 1.8 (p = 0.027), respectively, between the start and the end of the study. However, after Bonferroni correction, none of the directional changes in relative prevalence were statistically significant. In summary, there was no directional change in the relative prevalences between the start and the end of the study.

### Stability of the relative prevalences of the oMG strains over time

The relative prevalences of the oMG strains were stable over time in both *B. afzelii* and *B. garinii* (Figures S1, S2, S3 and S4). There were 110 proportion tests for *B. afzelii* (10 oMGs * 11 years) and 99 proportion tests for *B. garinii* (9 oMGs * 11 years). Assuming a type I error rate of 0.05, we expect that there should be 0.05 * 209 = 10.45 proportion tests that are statistically significant for the two *Borrelia* species. Of the 209 proportion tests, only 11 annual prevalences were significantly different (p < α = 0.05) from the average prevalence over the duration of the study (these years are marked with an asterisk in Figures S1, S2, S3 and S4). These 11 significant prevalences occurred in 6 different years and were distributed over 9 different oMG strains. The observed number of significant prevalences (11) was equal to the expected number of type I errors (10.45). This result supports the idea that the oMG strains did not fluctuate over time (Figures S3 and S4).

### Relationship between the R_0_-values and the relative prevalences of the oMG strains in *B. afzelii*

**Figure 3:**
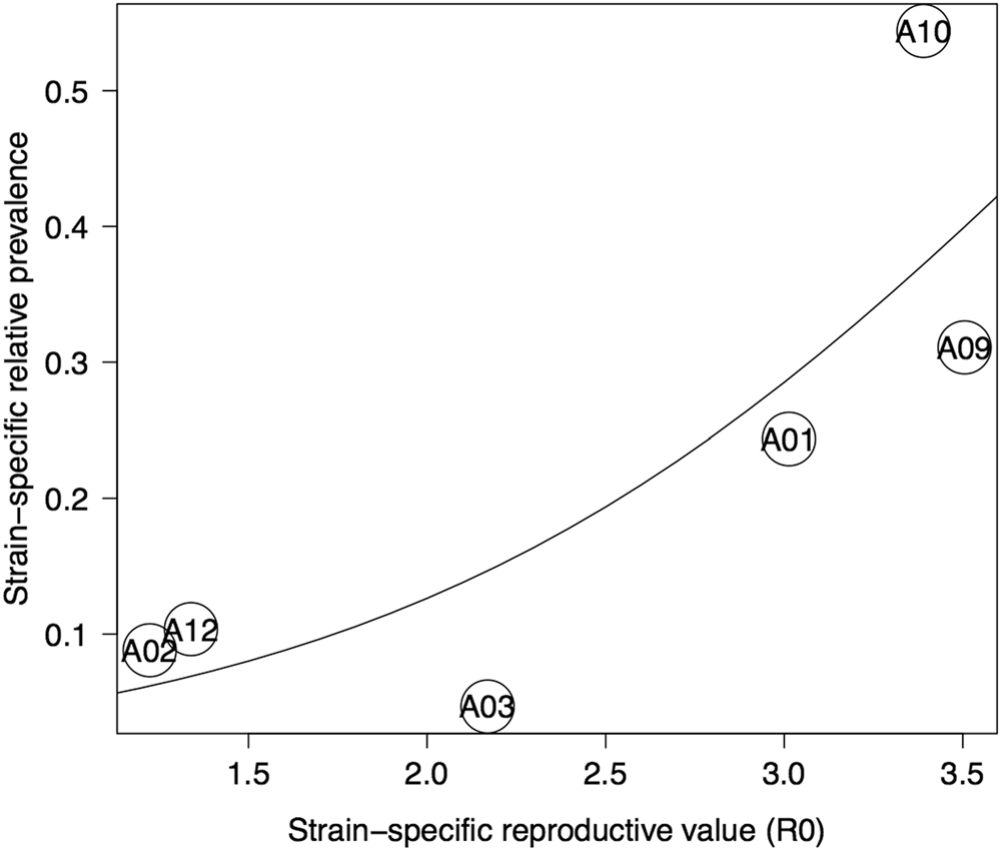
The reproductive numbers (R_0_) of six *B. afzelii* oMG strains determine the strain-specific relative prevalences in a wild population of *I. ricinus* nymphs. Each data point is labeled with the name of the oMG of the *B. afzelii* strain. The strain-specific R_0_ values were estimated from an experimental infection study using laboratory mice^43^. The strain-specific relative prevalences were estimated over the 11-year duration of the present study. The relative prevalence of an oMG strain is defined as the proportion of *B. afzelii*-infected nymphs that are infected with that particular oMG strain.

For the six *B. afzelii* oMG strains for which we had data^43^, there was a positive relationship between the strain-specific reproduction number (R_0_) in laboratory mice and the strain-specific relative prevalence in the questing *I. ricinus* nymphs (GLM with binomial errors; χ^2^ = 135.25, dof = 1, p < 0.001; Fig. 3). After correcting for overdispersion, the relationship remained statistically significant (GLM with quasibinomial errors; F_1, 4_ = 10.019, p = 0.034). The strain-specific R_0_ value explained 70.24% of the variation in the strain-specific relative prevalences (Fig. 3). Thus estimates of R_0_ using laboratory mice were a good predictor of the relative prevalences of the *B. afzelii* oMG strains in a wild population of *I. ricinus* nymphs.

## Discussion

The most interesting result from this study was that our estimates of R_0_ for six oMG strains of *B. afzelii* explained ~70% of the differences in prevalences among these strains in the field (Fig. 3). The strain-specific estimates of R_0_ were calculated using the three most important fitness components for any vector-borne pathogen: vector-to-host transmission, systemic (host-to-vector) transmission, and co-feeding transmission. These pathogen fitness components, in turn, were estimated using a laboratory Lyme borreliosis system that used *Mus musculus* mice as the reservoir host and *Ixodes ricinus* ticks as the vector. Thus laboratory estimates of transmission combined into a single measure of fitness (R_0_) were able to predict the community structure of this multiple-strain tick-borne pathogen in nature. Previous studies have used next generation matrix methods to estimate and compare R_0_ values between different tick-borne pathogens (e.g. *Borrelia burgdorferi* versus tick-borne encephalitis virus) to enhance our understanding of their ecology and epidemiology^18, 19, 46, 47^. Our study is an important demonstration that laboratory estimates of R_0_ can predict the strain-specific prevalences of a vector-borne pathogen in the field.

We had previously tested the relationship between our laboratory estimates of R_0_ and the relative prevalences of the oMG strains in the field, but the p-values were not significant^43^. In that study^43^, the prevalences of the oMG strains were obtained from the study by Pérez *et al*.^33^, who had collected nymph-derived isolates from two field sites in Switzerland, which are different from the Bois de l’Hôpital site in the present study. The major improvements in the present study are as follows. First, the prevalences of the *B. afzelii* oMG strains are based on a larger sample size at a single site, whereas the study by Pérez *et al*.^33^ had smaller sample sizes at two different sites. Second, we used 454-sequencing and the prevalences of the *B. afzelii* oMG strains were based on 114,432 *ospC* sequences, whereas in the study by Pérez *et al*.^33^, the prevalences of the oMG strains were based on cold single-strand conformational polymorphism analysis. We have previously pointed out that the sensitivity of single-strand conformational polymorphism analysis is much lower than that of 454-sequencing^21, 42^. For example, for the subset of *B. afzelii*-infected questing nymphs, Pérez *et al*.^33^ found that 1.5% carried multiple oMG strains, whereas we found that 78.8% carried multiple oMG strains^21^. Third, an implicit assumption underlying Fig. 3 is that the genetic backgrounds of the oMG strains used in the infection experiment of Tonetti *et al*.^43^ are similar to the oMG strains in our local tick population. This assumption is more likely to be met in the present study, because 3 of the 6 *B. afzelii* oMG strains (A2, A9, and A10) used to estimate the strain-specific R_0_ values in the infection experiment of Tonetti *et al*.^43^ came from the same Bois de l’Hôpital field site used to estimate the oMG strain prevalences in Fig. 3.

Studies on human pathogens consisting of multiple-strains such as *Neisseria meningitidis* and *Streptococcus pneumoniae* have shown that the frequencies of these strains can remain constant over time^14–16^. Similarly, we found that the community of oMG strains was stable over time and that some strains were consistently more common than others (Figs 2 and 3, S1 and S2). This result was particularly striking in *B. afzelii* where A10 was the most common oMG strain in 10 of the 11 years of the study (Fig. 2). We point out that the present study finds no evidence for the prediction found in the Lyme borreliosis literature that common oMG strains should decrease in frequency over time because they are preferentially targeted by the vertebrate immune system^22, 44, 45^. Previous studies on *B. burgdorferi* s. s. in *I. scapularis* ticks in the northeastern United States have documented rapid shifts in the prevalence distribution of the oMGs^17, 48, 49^. However, these studies were either done in tick populations where *B. burgdorferi* s. s. was emerging^48^ or over shorter time periods (3 years) using less reliable methods (single-strand conformational polymorphism analysis) for detecting multiple oMG strains in ticks^17, 49^. The present study of two sympatric *Borrelia* species is exceptional because of its long duration and because of its focus on a local population at a single small field site where Lyme borreliosis is endemic.

There are two hypotheses that explain how the *ospC* gene polymorphism of *B. burgdorferi* s. l. pathogens is maintained in nature: balancing or frequency-dependent selection^17, 35, 49, 50^ and multiple niche polymorphism^45, 51, 52^. In the frequency-dependent selection hypothesis, the vertebrate immune system is the mechanism that preferentially targets the most common strains and thereby prevents them from becoming too common^22, 35, 45, 50^. The OspC protein is an immunodominant antigen that induces a strong antibody response that protects the vertebrate host from secondary infection^53–57^. Protection is highly specific, and immunization with a given oMG antigen will only protect against infection with strains carrying the same oMG allele^58–60^. In summary, an immune-based explanation for the *ospC* polymorphism is intuitive because the OspC protein resembles other highly variable pathogen surface antigens that are under immune-based selection^61–64^.

Multiple niche polymorphism is the other hypothesis for the *ospC* polymorphism in *Borrelia* species^45, 51^. In this hypothesis, the different oMG strains of the *Borrelia* pathogen are adapted to different vertebrate host species, which represent different ecological niches^45, 51, 65^. The multiple niche polymorphism hypothesis was developed for *B. burgdorferi* s. s. in North America, which has a large host range^22, 23^, but the evidence for this hypothesis is conflicted^51, 52, 66^. *B. afzelii* has a much narrower host range than *B. burgdorferi* s. s. and is mostly associated with rodents^31, 33, 34^. Host blood meal analysis of *I. ricinus* ticks at our field site in Switzerland found that the most important rodent species were *Apodemus* mice, the bank vole (*Myodes glareolus*), and the red squirrel (*Sciurus vulgaris*)^67, 68^. With the exception of a system in France that contains an introduced species of chipmunk^40, 41^, other studies on *B. afzelii* in Europe have found little support for the multiple niche polymorphism hypothesis^33, 34, 39^. In the present study, the strain-specific estimates of R_0_ were based on a single rodent species (*Mus musculus*) but were still highly predictive of the strain structure in the field. This result shows that it was not necessary to consider multiple host species and provides an indirect argument against the multiple niche polymorphism hypothesis.

A number of reviews on the ecology of Lyme borreliosis have predicted that the frequencies of the oMG strains should cycle under frequency-dependent selection^22, 44, 45^. The present study found no evidence of cycles, but this result should not be interpreted as evidence against the frequency-dependent selection hypothesis. We point out here that immune-based models of selection on multiple-strain pathogens can produce all kinds of dynamics. Gupta and colleagues developed a number of theoretical models that explore how cross-reactive immune responses directed against immunodominant pathogen antigens (such as the OspC protein) influence the dynamics and community structure of multiple-strain pathogens^69–71^. A key finding of these models is that strong selection on immunodominant antigens by the host immune system will cause the pathogen population to organize into a set of unique serotypes that minimizes cross-reactive acquired immunity^71^. In such systems, the community of strains can be stable over long periods of time and the prevalence of each strain depends on its reproduction number (R_0_) as observed in the present study^69, 70^. With respect to another vector-borne parasite, the human malaria parasite *Plasmodium falciparum*, Gupta and colleagues suggested that, “many features of its epidemiology can be explained by assuming that it is a construct of ‘independently transmitted strains^69, 72^’”. Similarly, Qiu *et al*.^73^ suggested that the oMGs “could be viewed as evolutionarily stable “clonal complexes” within *B. burgdorferi* populations”. In summary, we suggest that immune-mediated selection on the OspC antigen is the best explanation for the maintenance of the *ospC* polymorphism in nature.

Due to its critical role in host invasion^74, 75^, the *ospC* gene has received much interest from a public health perspective. Genetic analysis of human isolates of *B. burgdorferi* s. l. revealed that only a subset of oMG strains is capable of infecting and causing disease in humans^4, 76, 77^. In the United States, oMG strains A, B, K, I, and N are most commonly associated with disseminated infections in humans but these strains are also the most common in questing *I. scapularis* ticks^76^. In the present study, strains carrying oMG A10 dominated the *B. afzelii* population in Neuchatel over the last decade (Fig. 1). Recent studies have shown that oMG strain A10 is common in other parts of Switzerland^33^ and Sweden^31^. In contrast, genetic screening of human isolates has never recovered oMG A10 from a human patient^36–38^. However, these genetic screens were based on a limited number of tissue biopsies (245 *ospC* sequences) that may have been sampled from areas in Europe where oMG A10 was not locally common^36–38^. Future studies should screen human isolates of *B. afzelii* to test whether strains carrying this oMG are infectious to humans.

In conclusion, our study on two common Lyme borreliosis pathogens in a local population of *I. ricinus* ticks showed that the community of *Borrelia* oMG strains was stable over a period of 11 years. In both *B. afzelii* and *B. garinii*, some oMG strains were consistently common whereas other oMG strains were consistently rare. In *B. afzelii*, the strain-specific estimates of R_0_ in laboratory rodents explained over 70% of the variation in the strain-specific prevalences in the field. Our results are consistent with theoretical models of how cross-reactive acquired immunity in the vertebrate host can determine the strain structure of pathogen populations^69–71^. The present study shows the importance of studying local pathogen populations over long periods of time to better understand their epidemiology.

## Methods

### Field sampling and molecular methods

The sampling of the *I. ricinus* ticks in the field, the testing for *Borrelia* infection, and the 454-sequencing of the single-copy *ospC* gene was described in a previous study^21^. Briefly, *I. ricinus* nymphs were sampled in a deciduous forest at the Bois de l’Hôpital site near the city of Neuchâtel (47°00′55.6″N, 6°94′16.7″E; surface of ~1 ha) over a period of 11 years (2000 to 2010). Nymphs were screened for spirochete infection using immunofluorescence microscopy and *Borrelia*-infected nymphs were incubated in BSK II medium at 34 °C. DNA was extracted from all spirochete-positive BSK cultures and the *Borrelia* species was identified using a PCR-reverse line blot assay that targets the 23S-5S spacer gene^78^. The experimental design was described in a previous study^21^. Only those isolates that were singly infected with *B. afzelii* or *B. garinii* were selected for 454-sequencing of the *ospC* gene. For each *Borrelia* species, a maximum of 20 isolates was randomly selected for each of the 11 years of the survey for a total of 193 *B. afzelii* isolates and 190 *B. garinii* isolates. For each of these 383 isolates, the *ospC* gene was amplified using the PCR protocol of Bunikis *et al*.^36^. 454-sequencing of the amplicons in the forward direction produced 240,410 useable *ospC* gene sequences (reads) and each sequence was 521 bp long. For each nymphal-tick derived isolate, the mean coverage was 632 *ospC* gene sequences (reads).

### Identification of the *ospC* major groups

The *ospC* gene sequences can be classified into what are called *ospC* major groups (oMGs). The oMGs have a highly discrete pattern of genetic variation where each oMG is ≥8% different in DNA sequence from all other oMGs^35–38, 42, 51^. For the three Lyme borreliosis pathogens that have been most studied to date, *B. burgdorferi* s. s., *B. afzelii*, and *B. garinii*, each *Borrelia* species contains ~20 oMGs worldwide, with local populations often having 50% or more of this diversity^17, 21, 35, 36, 38, 40–42, 51^. We had previously shown that all 240,410 *ospC* gene sequences clustered into 23 distinct oMGs that were 8% divergent from each other in DNA sequence^21^. Within each oMG, the DNA sequence variation was <2%^21^. We did not find any *ospC* gene sequences that were intermediately divergent (2–8%)^21^. This finding is important because it shows that the oMG alleles are real biological categories that are relatively robust to errors in sequencing or to changes in the clustering protocol. For example, changing the similarity threshold of our clustering protocol from 93–98% did not affect the number of unique oMGs in our dataset^21^.

### Nomenclature of the oMGs of *B. afzelii* and *B. garinii*

In the literature, there are two different nomenclatures for the oMGs of *B. afzelii* and *B. garinii* ^36, 38^. In the present study, we used the nomenclature system that was developed by Bunikis *et al*.^36^, and which has been used by others and we^21, 31, 34, 42^. According to this nomenclature system, the 23 oMGs were as follows: 10 for *B. afzelii* (A1, A2, A3, A5, A7, A9, A10, A11, A12, and A14), 11 for *B. garinii* (G2, G4, G6, G7, G8, G9, G10, G11, G13, G14, and G15), 1 for *B. burgdorferi* s. s. (Q), and 1 for *B. valaisiana* (V1)^21^. The present study is restricted to the oMGs belonging to *B. afzelii* and *B. garinii*. In what follows, we will refer to a tick-derived isolate carrying a particular oMG allele as an oMG strain.

### Calculation of the relative prevalences of the oMG strains over time

The community of oMG strains was determined for each of the 383 nymph-derived spirochete isolates as described previously^21^. An oMG was considered as present as long as the isolate contained a single sequence belonging to that group^21^. For each oMG strain, the annual relative prevalence was calculated as the proportion of nymph-derived spirochete isolates that carried that particular oMG that year^21^. In our previous study, we investigated patterns of oMG strain diversity in ticks and patterns of co-occurrence of oMG strains within ticks^21^. Those analyses combined all the data over the 11 years of the study and ignored temporal variation in the prevalences of the oMG strains, which is the focus of the present study.

### Important assumptions of this study

This study makes three important assumptions. The first assumption is that the *ospC* gene is a reliable genetic marker for a given strain. The second assumption is that the step of culturing the *Borrelia* isolates in BSK media did not change the composition of the oMG strains. The third assumption is that the PCR protocol used to amplify the *ospC* gene was equally effective at amplifying all of the different oMG alleles. We address each of these three assumptions in the supplementary information.

## Statistical Methods

### Directional change in the relative prevalences of the oMG strains over time

To visualize whether the community of oMG strains changed between the first and second halves of the study, we compared the relative prevalences of the oMGs for the first six years (2000 to 2005) and for the last five years of the study (2006 to 2010). We used a Pearson’s correlation test to determine whether there was a correlation in the relative prevalences of the oMG strains between these two periods of time. This test was done separately for *B. afzelii* and *B. garinii*. We used a proportion test to determine whether the relative prevalence of any of the oMG strains had changed between the first six years (2000 to 2005) and the last five years of the study (2006 to 2010). This approach allows us to detect large directional changes in relative prevalence over the duration of the study but not scenarios where the prevalences of the strains cycle within the 11-year period of the study.

### Stability of the relative prevalences of the oMG strains over time

Previous reviews suggested that the oMG strains should cycle over time^22, 44, 45^. If true, we would expect the years where a given strain is common or rare to deviate significantly from the long-term average of that strain over the course of the study. For each of the 10 *B. afzelii* oMGs (A1, A2, A3, A5, A7, A9, A10, A11, A12, and A14), we used a proportion test to determine whether its relative prevalence in a given year was significantly different from the average long-term relative prevalence of that strain over the duration of the study. We did the same for each of the 9 most common *B. garinii* oMGs (G2, G4, G6, G7, G8, G9, G11, G13, and G14; oMGs G10 and G15 were too rare to be analysed). To test for the stability of the relative prevalences of the oMG strains, we summed the number of significant deviations from the long-term average for all the oMG strains belonging to the same *Borrelia* species. The observed number of significant deviations was then compared to the null hypothesis that deviations were caused by random binomial sampling error.

### Relationship between R_0_ and the relative prevalences of the oMG strains

The R_0_ of an infection can be thought of as the number of cases one case generates, on average, over the course of its infectious period, in an otherwise uninfected population. In the absence of immunity-mediated competition between strains, theory predicts that the R_0_ value of each strain will determine its prevalence in nature^70^. We recently conducted a study where laboratory mice were experimentally infected via tick bite with one of six *B. afzelii* oMG strains: A1, A2, A3, A9, A10, and A12^43^. To avoid confusion, we point out that Tonetti *et al*.^43^ used the nomenclature developed by Lagal *et al*.^38^ and Pérez *et al*.^33^ and the six *B. afzelii* oMG strains in that study are therefore referred to as A2, ME, A3, A1, YU, and A4, respectively. The purity of these six strains was recently confirmed by 454-sequencing of the *ospC* gene (Table S1). Importantly, three of these isolates, A2, A9, and A10, were obtained from the Neuchâtel area, whereas isolates A1, A3, and A12 were obtained from Thune (Switzerland), Austria, and Germany respectively. For each of the six strains, the following three fitness components were measured: tick-to-host transmission, systemic (host-to-tick) transmission, and co-feeding transmission^43^.

We used next generation matrix methods^18, 46, 47^ to combine these transmission components into the reproduction number (R_0_) for each of the six *B. afzelii ospC* strains^43^. The study of Tonetti *et al*.^43^ assumed that the efficiency of vertical transmission (*r*_*A*_) was 0.10 and that the proportion of competent hosts (*h*_*c*_) was 0.50. Recent work suggests that transovarial transmission of *B. burgdorferi* s. l. does not occur in *Ixodes* ticks^79, 80^ and the value of *r*_*A*_ was therefore set to 0.00 in the present study. Host blood meal analysis in our local Lyme borreliosis system suggests that only 28.0% of questing immature *I. ricinus* ticks obtained their blood meal from *B. afzelii*-competent rodent reservoir hosts^68^, and the value of *h*_*c*_ was therefore set to 0.28 in the present study. Thus the estimates of R_0_ for the six strains of *B. afzelii* are similar but not identical between Tonetti *et al*.^43^ and the present study. We used a generalized liner model (GLM) with binomial errors to test whether there was a positive relationship between the R_0_ values of the six *B. afzelii ospC* strains and the strain-specific relative prevalences in the local *I. ricinus* population (averaged over the entire course of the study). We calculated the associated r^2^ using the McFadden’s pseudo r^2^ method.

## Electronic supplementary material

Supplementary information

Supplementary data

## References

[1] Balmer O, Tanner M (2011). Prevalence and implications of multiple-strain infections. Lancet Infectious Diseases.

[2] Schmid-Hempel, P. *Evolutionary Parasitology: The Integrated Study of Infections, Immunology, Ecology, and Genetics*. (Oxford University Press, 2011).

[3] Alizon S, de Roode JC, Michalakis Y (2013). Multiple infections and the evolution of virulence. Ecol. Lett..

[4] Seinost G (1999). Four clones of *Borrelia burgdorferi* sensu stricto cause invasive infection in humans. Infect. Immun..

[5] Yazdankhah SP (2004). Distribution of serogroups and genotypes among disease-associated and carried isolates of *Neisseria meningitidis* from the Czech Republic, Greece, and Norway. J. Clin. Microbiol..

[6] Gottlieb GS (2004). Dual HIV-1 infection associated with rapid disease progression. Lancet.

[7] Henning L (2004). A prospective study of Plasmodium falciparum multiplicity of infection and morbidity in Tanzanian children. Trans. R. Soc. Trop. Med. Hyg..

[8] Mueller I (2012). Force of infection is key to understanding the epidemiology of *Plasmodium falciparum* malaria in Papua New Guinean children. Proc. Natl. Acad. Sci. USA.

[9] Lipsitch M, O’Hagan JJ (2007). Patterns of antigenic diversity and the mechanisms that maintain them. J. Royal Soc. Interface.

[10] Wargo AR, Huijben S, de Roode JC, Shepherd J, Read AF (2007). Competitive release and facilitation of drug-resistant parasites after therapeutic chemotherapy in a rodent malaria model. Proc. Natl. Acad. Sci. USA.

[11] Read AF, Day T, Huijben S (2011). The evolution of drug resistance and the curious orthodoxy of aggressive chemotherapy. Proc. Natl. Acad. Sci. USA.

[12] Pollitt, L. C. *et al*. Rapid response to selection, competitive release and increased transmission potential of artesunate-selected *Plasmodium chabaudi* malaria parasites. *PLOS Pathog*. **10** (2014). 10.1371/journal.ppat.1004019 PMC3999151 24763470

[13] Read, A. F. & Mackinnon, M. In *Evolution in Health and* Disease (eds Stearns, S. C. & Koella, J. C.) (Oxford University Press, 2007).

[14] Buckee CO, Gupta S, Kriz P, Maiden MCJ, Jolley KA (2010). Long-term evolution of antigen repertoires among carried meningococci. P. Roy. Soc. B-Biol. Sci..

[15] Bambini, S. *et al*. An analysis of the sequence variability of meningococcal fHbp, NadA and NHBA over a 50-year period in the Netherlands. *PLOS ONE* **8** (2013). 10.1371/journal.pone.0065043 PMC3663754 23717687

[16] Weinberger, D. M. *et al*. Pneumococcal capsular polysaccharide structure predicts serotype prevalence. *PLOS Pathog*. **5** (2009). 10.1371/journal.ppat.1000476 PMC2689349 19521509

[17] Qiu WG, Dykhuizen DE, Acosta MS, Luft BJ (2002). Geographic uniformity of the Lyme disease spirochete (*Borrelia burgdorferi*) and its shared history with tick vector (*Ixodes scapularis*) in the northeastern United States. Genetics.

[18] Hartemink NA, Randolph SE, Davis SA, Heesterbeek JAP (2008). The basic reproduction number for complex disease systems: Defining R-0 for tick-borne infections. Am. Nat..

[19] Matser A, Hartemink N, Heesterbeek H, Galvani A, Davis S (2009). Elasticity analysis in epidemiology: an application to tick-borne infections. Ecol. Lett..

[20] Juliano JJ (2010). Exposing malaria in-host diversity and estimating population diversity by capture-recapture using massively parallel pyrosequencing. Proc. Natl. Acad. Sci. USA.

[21] Durand J (2015). Cross-immunity and community structure of a multiple-strain pathogen in the tick vector. Appl. Environ. Microbiol..

[22] Kurtenbach, K. *et al*. Fundamental processes in the evolutionary ecology of Lyme borreliosis. *Nat. Rev. Microbiol*. **4** (2006). 10.1038/nrmicro1475 16894341

[23] Piesman J, Gern L (2004). Lyme borreliosis in Europe and North America. Parasitology.

[24] Gern, L. & Humair, P.-F. In *Lyme borreliosis: biology, epidemiology, and control* 149-174 (CABI Publishing, 2002).

[25] Kurtenbach K (2002). Host association of *Borrelia burgdorferi* sensu lato - the key role of host complement. Trends Microbiol..

[26] Heylen DJA (2017). Inefficient co-feeding transmission of Borrelia afzelii in two common European songbirds. Scientific Reports.

[27] Herrmann C, Gern L, Voordouw M (2013). Species co-occurrence patterns among Lyme borreliosis pathogens in the tick vector *Ixodes ricinus*. Appl. Environ. Microbiol..

[28] Kurtenbach K (2001). Distinct combinations of *Borrelia burgdorferi* sensu lato genospecies found in individual questing ticks from Europe. Appl. Environ. Microbiol..

[29] Gern L, Douet V, Lopez Z, Rais O, Moran Cadenas F (2010). Diversity of *Borrelia* genospecies in *Ixodes ricinus* ticks in a Lyme borreliosis endemic area in Switzerland identified by using new probes for reverse line blotting. Ticks Tick Borne Dis..

[30] Rauter C, Hartung T (2005). Prevalence of *Borrelia burgdorferi* sensu lato genospecies in *Ixodes ricinus* ticks in Europe: a metaanalysis. Appl. Environ. Microbiol..

[31] Andersson M, Scherman K, Raberg L (2013). Multiple-strain infections of *Borrelia afzelii*: a role for within-host interactions in the maintenance of antigenic diversity?. Am. Nat..

[32] Heylen D, Matthysen E, Fonville M, Sprong H (2014). Songbirds as general transmitters but selective amplifiers of *Borrelia burgdorferi* sensu lato genotypes in *Ixodes rinicus* ticks. Environ. Microbiol..

[33] Pérez D, Kneubühler Y, Rais O, Jouda F, Gern L (2011). *Borrelia afzelii ospC* genotype diversity in *Ixodes ricinus* questing ticks and ticks from rodents in two Lyme borreliosis endemic areas: Contribution of co-feeding ticks. Ticks Tick Borne Dis..

[34] Strandh, M. & Raberg, L. Within-host competition between *Borrelia afzelii ospC* strains in wild hosts as revealed by massively parallel amplicon sequencing. *Philos. T. Roy. Soc. B* **370** (2015). 10.1098/rstb.2014.0293 PMC4528491 26150659

[35] Wang IN (1999). Genetic diversity of *ospC* in a local population of *Borrelia burgdorferi* sensu stricto. Genetics.

[36] Bunikis J (2004). Sequence typing reveals extensive strain diversity of the Lyme borreliosis agents *Borrelia burgdorferi* in North America and *Borrelia afzelii* in Europe. Microbiology-Sgm.

[37] Baranton G, Seinost G, Theodore G, Postic D, Dykhuizen D (2001). Distinct levels of genetic diversity of *Borrelia burgdorferi* are associated with different aspects of pathogenicity. Res. Microbiol..

[38] Lagal V, Postic D, Ruzic-Sabljic E, Baranton G (2003). Genetic diversity among *Borrelia* strains determined by single-strand conformation polymorphism analysis of the *ospC* gene and its association with invasiveness. J. Clin. Microbiol..

[39] Hellgren O, Andersson M, Raberg L (2011). The genetic structure of *Borrelia afzelii* varies with geographic but not ecological sampling scale. J. Evol. Biol..

[40] Jacquot M (2014). High-throughput sequence typing reveals genetic differentiation and host specialization among populations of the *Borrelia burgdorferi* species complex that infect rodents. PLOS ONE.

[41] Jacquot, M. *et al*. Multiple independent transmission cycles of a tick-borne pathogen within a local host community. *Scientific Reports* **6** (2016). 10.1038/srep31273 PMC4976386 27498685

[42] Durand, J. *et al*. Multi-strain infections of the Lyme borreliosis pathogen in the tick vector. *Appl. Environ. Microbiol*. **83** (2016). 10.1128/AEM.02552-16 PMC5244308 27836839

[43] Tonetti N, Voordouw MJ, Durand J, Monnier S, Gern L (2015). Genetic variation in transmission success of the Lyme borreliosis pathogen *Borrelia afzelii*. Ticks Tick Borne Dis..

[44] Tsao, J. Reviewing molecular adaptations of Lyme borreliosis spirochetes in the context of reproductive fitness in natural transmission cycles. *Vet. Res*. **40** (2009). 10.1051/vetres/2009019 PMC2701186 19368764

[45] Brisson D, Drecktrah D, Eggers C, Samuels DS (2012). Genetics of *Borrelia burgdorferi*. Annu. Rev. Genet..

[46] Harrison A, Montgomery WI, Bown KJ (2011). Investigating the persistence of tick-borne pathogens via the R-0 model. Parasitology.

[47] Harrison A, Bennett N (2012). The importance of the aggregation of ticks on small mammal hosts for the establishment and persistence of tick-borne pathogens: an investigation using the R-0 model. Parasitology.

[48] MacQueen D (2012). Genotypic diversity of an emergent population of *Borrelia burgdorferi* at a coastal Maine island recently colonized by *Ixodes scapularis*. Vector Borne and Zoonotic Diseases.

[49] Qiu WG (1997). A population genetic study of *Borrelia burgdorferi* sensu stricto from eastern Long Island, New York, suggested frequency-dependent selection, gene flow and host adaptation. Hereditas.

[50] Dykhuizen DE, Baranton G (2001). The implications of a low rate of horizontal transfer in *Borrelia*. Trends Microbiol..

[51] Brisson D, Dykhuizen DE (2004). *ospC* diversity in *Borrelia burgdorferi*: different hosts are different niches. Genetics.

[52] Vuong HB (2014). Occurrence and transmission efficiencies of *Borrelia burgdorferi ospC* types in avian and mammalian wildlife. Infect. Genet. Evol..

[53] Dressler F, Whalen JA, Reinhardt BN, Steere AC (1993). Western blotting in the serodiagnosis of Lyme disease. J. Infect. Dis..

[54] Engstrom SM, Shoop E, Johnson RC (1995). Immunoblot interpretation criteria for serodiagnosis of early Lyme disease. J. Clin. Microbiol..

[55] Fung BP, McHugh GL, Leong JM, Steere AC (1994). Humoral immune response to outer surface protein C of *Borrelia burgdorferi* in Lyme disease: role of the immunoglobulin M response in the serodiagnosis of early infection. Infect. Immun..

[56] Mbow ML, Gilmore RD, Titus RG (1999). An OspC-specific monoclonal antibody passively protects mice from tick-transmitted infection by *Borrelia burgdorferi* B31. Infect. Immun..

[57] Barthold SW (1999). Specificity of infection-induced immunity among *Borrelia burgdorferi* sensu lato species. Infect. Immun..

[58] Jacquet M, Durand J, Rais O, Voordouw MJ (2015). Cross-reactive acquired immunity influences transmission success of the Lyme disease pathogen, *Borrelia afzelii*. Infect. Genet. Evol..

[59] Probert WS, Crawford M, Cadiz RB, LeFebvre RB (1997). Immunization with outer surface protein (Osp) A, but not OspC, provides cross-protection of mice challenged with North American isolates of *Borrelia burgdorferi*. J. Infect. Dis..

[60] Earnhart CG, Buckles EL, Dumler JS, Marconi RT (2005). Demonstration of OspC type diversity in invasive human Lyme disease isolates and identification of previously uncharacterized epitopes that define the specificity of the OspC murine antibody response. Infect. Immun..

[61] Conway DJ, Polley SD (2002). Measuring immune selection. Parasitology.

[62] Abal-Fabeiro JL, Maside X, Bello X, Llovo J, Bartolome C (2013). Multilocus patterns of genetic variation across *Cryptosporidium* species suggest balancing selection at the gp60 locus. Mol. Ecol..

[63] Polley SD, Conway DJ (2001). Strong diversifying selection on domains of the *Plasmodium falciparum* apical membrane antigen 1 gene. Genetics.

[64] Urwin R (2004). Distribution of surface protein variants among hyperinvasive meningococci: Implications for vaccine design. Infect. Immun..

[65] Hanincova K, Kurtenbach K, Diuk-Wasser M, Brei B, Fish D (2006). Epidemic spread of Lyme borreliosis, Northeastern United States. Emerg. Infect. Dis..

[66] Mechai, S. *et al*. Evidence for host-genotype associations of *Borrelia burgdorferi* sensu stricto. *PLOS ONE* **11** (2016). 10.1371/journal.pone.0149345 PMC4763156 26901761

[67] Humair P-F (2007). Molecular identification of bloodmeal source in *Ixodes ricinus* ticks using 12S rDNA as a genetic marker. J. Med. Entomol..

[68] Morán Cadenas FM (2007). Identification of host bloodmeal source and *Borrelia burgdorferi* sensu lato in field-collected *Ixodes ricinus* ticks in Chaumont (Switzerland). J. Med. Entomol..

[69] Gupta S, Anderson RM (1999). Population structure of pathogens: The role of immune selection. Parasitol. Today.

[70] Gupta S, Ferguson N, Anderson R (1998). Chaos, persistence, and evolution of strain structure in antigenically diverse infectious agents. Science.

[71] Gupta S (1996). The maintenance of strain structure in populations of recombining infectious agents. Nature Medicine.

[72] Gupta S, Trenholme K, Anderson RM, Day KP (1994). Antigenic diversity and the transmission dynamics of *Plasmodium falciparum*. Science.

[73] Qiu WG (2004). Genetic exchange and plasmid transfers in *Borrelia burgdorferi* sensu stricto revealed by three-way genome comparisons and multilocus sequence typing. Proc. Natl. Acad. Sci. USA.

[74] Grimm D (2004). Outer-surface protein C of the Lyme disease spirochete: a protein induced in ticks for infection of mammals. Proc. Natl. Acad. Sci. USA.

[75] Tilly K (2006). *Borrelia burgdorferi* OspC protein required exclusively in a crucial early stage of mammalian infection. Infect. Immun..

[76] Dykhuizen DE (2008). Short report: The propensity of different *Borrelia burgdorferi* sensu stricto genotypes to cause disseminated infections in humans. Am. J. Trop. Med. Hyg..

[77] Wormser GP (2008). *Borrelia burgdorferi* genotype predicts the capacity for hematogenous dissemination during early Lyme disease. J. Infect. Dis..

[78] Burri C, Cadenas FM, Douet V, Moret J, Gern L (2007). *Ixodes ricinus* density and infection prevalence of *Borrelia burgdorferi* sensu lato along a north-facing altitudinal gradient in the Rhone Valley (Switzerland). Vector-Borne Zoonot..

[79] Rollend L, Fish D, Childs JE (2013). Transovarial transmission of *Borrelia* spirochetes by *Ixodes scapularis*: A summary of the literature and recent observations. Ticks Tick Borne Dis..

[80] Richter D, Debski A, Hubalek Z, Matuschka FR (2012). Absence of Lyme disease spirochetes in larval *Ixodes ricinus* ticks. Vector-Borne Zoonot..

