# Supplementary material for: Fitness estimates from experimental infections predict the long-term strain structure of a vector-borne pathogen in the field
Source: Sci Rep. 2017 May 12;7:1851. doi: 10.1038/s41598-017-01821-1 (PMC5431797; doi:10.1038/s41598-017-01821-1)
Supplement: Supplementary file 1 — Supplementary information [file 41598_2017_1821_MOESM1_ESM.pdf]

Supplementary information

Title: Fitness estimates from experimental infections predict the long-term strain structure of a vector-borne pathogen in the field

Authors: Jonas Durand, Maxime Jacquet, Olivier Rais, Lise Gern, Maarten J. Voordouw

Important assumption of the *ospC*-typing system: An important assumption in all studies that use the *ospC*-typing system [1-5] is that the *ospC* gene is a reliable genetic marker for a given strain. Thus, we assume that the oMG alleles are consistently associated with genetic variation at other *Borrelia* loci that determine the prevalences of the oMG strains in our local tick population over the duration of the study. Previous work has shown that there is substantial recombination at the *ospC* locus both within and between *Borrelia* species [6-8]. For this reason, the *ospC* gene is not suitable for phylogenetic studies that reconstruct the evolutionary relationships between the different *Borrelia* species [7,8]. However, studies of local populations of *B. burgdorferi* s. s. and *B. afzelii* have found strong linkage disequilibrium between the oMGs and multi-locus sequence types based on other *Borrelia* genes (e.g. chromosomal housekeeping genes or polymorphic plasmid-encoded genes) [6,9,10]. Counter-intuitively, this linkage disequilibrium is often stronger at smaller geographic scales [3]. For example, linkage disequilibrium between the oMGs and the multi-locus sequence types based on 8 chromosomal housekeeping genes was nearly perfect for 24 strains of *B. burgdorferi* s. s. sampled from Westchester County, New York State [11]. In contrast, at larger spatial scales (e.g. New York versus California), the same set of oMGs was associated with a different sets of multi-locus sequence types [11]. In summary, the oMG is a reliable genetic marker for differentiating among strains of the same *Borrelia* species at small spatial scales [3,9,10].

Important assumption of genotyping cultures of *Borrelia* strains: In the present study, we used nymph-derived *Borrelia* isolates that were grown up in BSK culture. One concern was that the BSK culture step induced selection on the community of oMG strains. To address this concern, we analysed the community of oMG strains in an additional sample of 253 *Borrelia*-infected *I. ricinus* nymphs where the DNA was extracted directly from the ticks without an intervening culture step [5]. Of these 253 nymphs, 152 and 101 were infected with *B. afzelii* and *B. garinii*, respectively [5]. These nymphs had been collected from the same field site over a period of three years (2009 to 2011) as part of the PhD thesis of Coralie Herrmann. Details of the field sampling and subsequent molecular methods have been described previously [5,12]. We used the same 454-sequencing protocol to determine the community of oMG strains in each nymph [5]. To test if there was an effect of the origin of the DNA extract (cultured isolate versus nymphal tick) on the community of oMG strains, we focused on the two years of overlap between the two studies (2009, 2010). We used Pearson correlations to compare the relative prevalences of the oMG strains between the two studies for each of the two *Borrelia* species.

There was a strong correlation in the prevalences of the oMG strains (data were combined for 2009 and 2010) between the nymph-derived *Borrelia* isolates [1] and the nymph-derived DNA extracts in both *B. afzelii* (Pearson correlation test: $r = 0.845$, $t = 7.056$, $df = 20$, $p < 0.001$; Figure S5) and *B. garinii* ($r = 0.516$, $t = 2.559$, $df = 18$, $p = 0.019$; Figure S5). Thus the step of culturing nymph-derived isolates in BSK medium did not induce strong selection on the composition of the community of oMG strains. We also note that previous work had shown that the ability to detect *B. burgdorferi* s. l. infections in ticks is actually higher for the method of BSK culture followed by DNA extraction than direct DNA extraction of ticks [13].

Important assumption of the efficacy of PCR amplification: Another important assumption is that the PCR protocol used to amplify the *ospC* gene was equally effective at

amplifying all of the different oMG alleles. We used the same *ospC* primers as Strandh and Raberg [4], who tested this assumption and showed that there was no amplification bias. Thus we are confident that the results in the present study were not biased by the BSK culture of the *Borrelia* isolates or PCR bias for certain oMG alleles.

Table S1. The purity with respect to the *ospC* gene is shown for the six *B. afzelii* isolates used in the experimental infection study by Tonetti et al. [14]. These six *B. afzelii* isolates were used to calculate the strain-specific fitness (R_0) in laboratory mice. The dominant *ospC* major group (oMG) is shown for each isolate. The *ospC* gene of each of the six isolates was sequenced using 454 sequencing.

Isolate	oMG	Total number of sequences	Purity
NE36	A1	873	99.20%
NE4054	A2	141	99.29%
E61	A3	977	100.00%
NE5046	A9	131	99.24%
NE4049	A10	1313	100.00%
NE4051	A10	162	100.00%
P/sto	A12	1105	99.70%

Table S2. Directional changes in the relative prevalences between the start and the end of the study are shown for each *ospC* major group (oMG). We used a proportion test to determine whether the relative prevalence of any of the oMGs had changed between the first six years (2000 to 2005) and the last five years of the study (2006 to 2010).

species	*ospC*	inf1/total1	inf2/total2	p.inf1	p.inf2	chi.sq	signif
afzelii	A1	27/97	20/96	0.278	0.208	0.932	0.334
afzelii	A2	9/97	8/96	0.093	0.083	0.000	1.000
afzelii	A3	2/97	7/96	0.021	0.073	1.909	0.167
afzelii	A5	4/97	11/96	0.041	0.115	2.670	0.102
afzelii	A7	4/97	10/96	0.041	0.104	1.982	0.159
afzelii	A9	24/97	36/96	0.247	0.375	3.094	0.079
afzelii	A10	56/97	49/96	0.577	0.510	0.622	0.430
afzelii	**A11**	**4/97**	**16/96**	**0.041**	**0.167**	**6.877**	**0.009**
afzelii	A12	13/97	7/96	0.134	0.073	1.337	0.248
afzelii	**A14**	**17/97**	**31/96**	**0.175**	**0.323**	**4.868**	**0.027**

species	*ospC*	inf1/total1	inf2/total2	p.inf1	p.inf2	chi.sq	signif
garinii	G2	21/104	19/86	0.202	0.221	0.020	0.888
garinii	G4	15/104	13/86	0.144	0.151	0.000	1.000
garinii	G6	11/104	11/86	0.106	0.128	0.061	0.805
garinii	G7	36/104	23/86	0.346	0.267	1.019	0.313
garinii	G8	47/104	33/86	0.452	0.384	0.640	0.424
garinii	G9	24/104	19/86	0.231	0.221	0.000	1.000
garinii	G10	2/104	1/86	0.019	0.012	0.000	1.000
garinii	G12	20/104	8/86	0.192	0.093	2.945	0.086
garinii	G13	33/104	20/86	0.317	0.233	1.286	0.257
garinii	G14	32/104	25/86	0.308	0.291	0.009	0.924
garinii	G15	6/104	1/86	0.058	0.012	1.666	0.197

inf = number of nymphs infected with that particular oMG
total = number of nymphs infected with *B. afzelii*
p.inf = prevalence = proportion of infected nymphs (inf/total)
subscript 1 = the first six years of the study (2000 to 2005)
subscript 2 = the last five years of the study (2006 to 2010)
chi.sq = Chi-square test statistic of the proportion test comparing p.inf1 and p.inf2
signif = Statistical significance of the proportion test comparing p.inf1 and p.inf2. Statistically significant p-values ($p < 0.05$) are shown in bold.

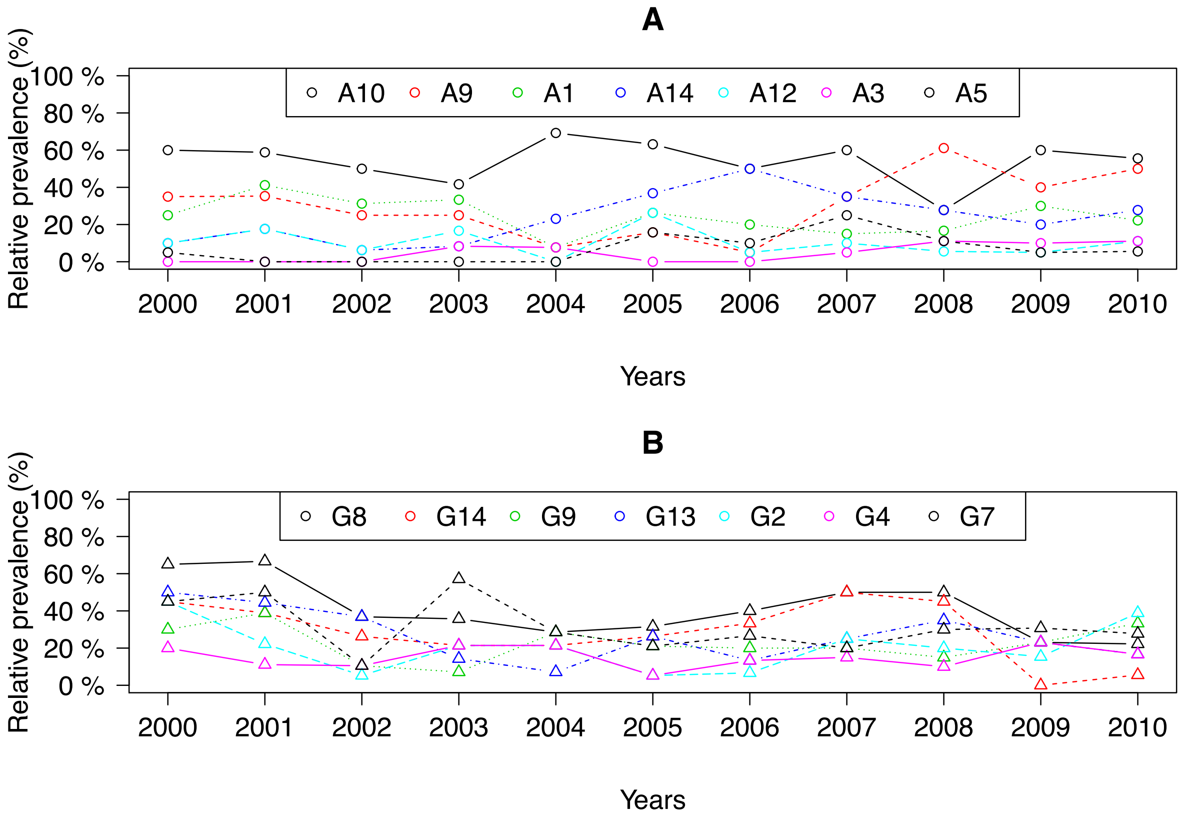

Figure S1. Relative prevalences of the *ospC* major groups are shown for *Borrelia afzelii* and *B. garinii* over the eleven years of the study. The relative prevalences are shown for the seven most common *ospC* major groups (oMGs) of (A) *B. afzelii* and (B) *B. garinii*. The relative prevalences were calculated as the proportion of nymphal tick-derived isolates infected with a particular oMG. The community of *B. afzelii* was dominated by strains carrying oMG A10 for the duration of the study. The relative prevalence of an oMG strain is defined as the proportion of *B. afzelii*-infected nymphs that are infected with that particular oMG strain.

Statistical Methods: To test whether the relative prevalences of the oMGs changed over time we used the following analysis. We calculated the Pearson's correlation of the relative prevalence distribution of the oMGs for each of the 55 pairs of years. We then tested whether the magnitude of this pairwise correlation decreased with the time interval between the two years in each pair.

Results: In addition, with respect to the annual relative prevalence distribution of the oMGs, the magnitude of the Pearson's correlation coefficient between years remained stable as the time interval increased between the years in the pair in both *B. afzelii* (Linear regression: $r^2 = 0.018$, $F = 0.018$, $p = 0.894$; Figure S2) and *B. garinii* (Linear regression: $r^2 = 0.015$, $F = 0.203$, $p = 0.654$; Figure S2).

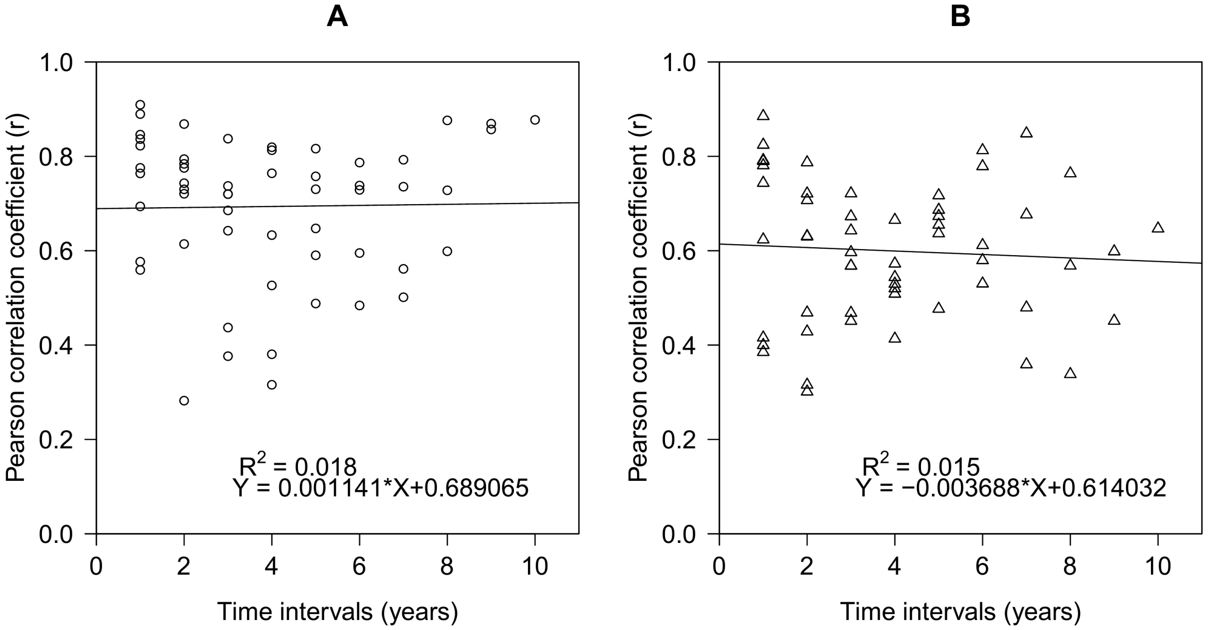

Figure S2. The relative prevalence distribution of the *ospC* major groups is stable over time. The Pearson correlation coefficient (r) of the relative prevalence distribution of the *ospC* major groups (oMGs) was calculated for each pair of years (n = 55 pairs). The relationship between these r-values and the time interval between the years in the pair is shown for both (A) *B. afzelii* and (B) *B. garinii*. For both *Borrelia* species, there is no significant relationship between r and the time interval showing that the relative prevalences of the different oMGs are stable over time. The r^2-value and the equation of the linear regression are indicated on each plot.

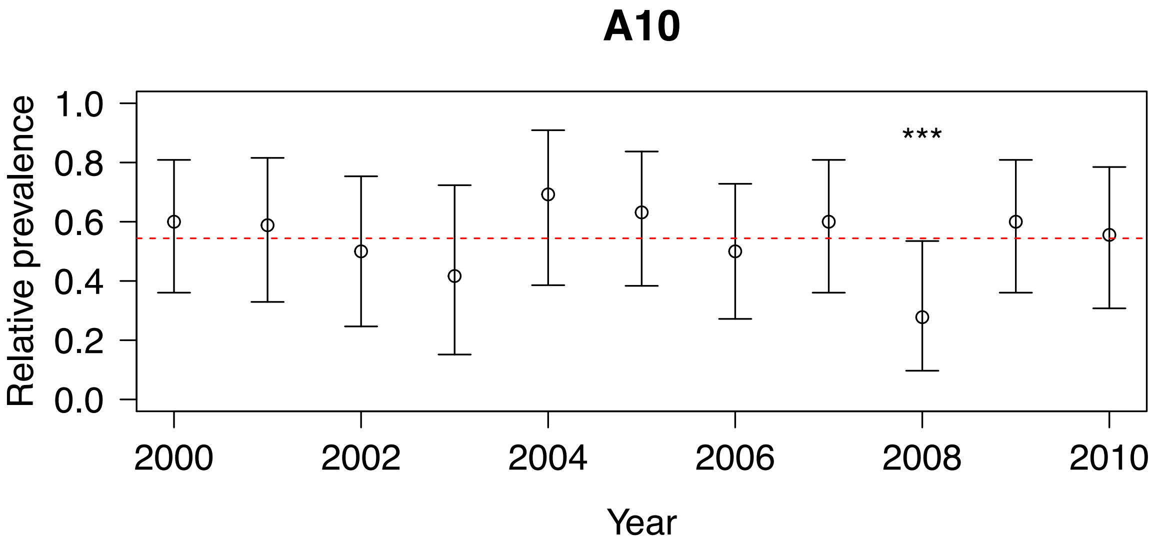

Figure S3a. The relative prevalences of *Borrelia afzelii* oMG A10 over the 11 years of the study. The relative prevalences were calculated as the proportion of nymphal tick-derived *B. afzelii* isolates infected with oMG A10. The bars indicate the 95% confidence interval and the red dotted line shows the mean relative prevalence of oMG A10 over the duration of the study. The asterisks indicate the years where the prevalences were significantly different ($\alpha = 0.05$) from the long-term mean.

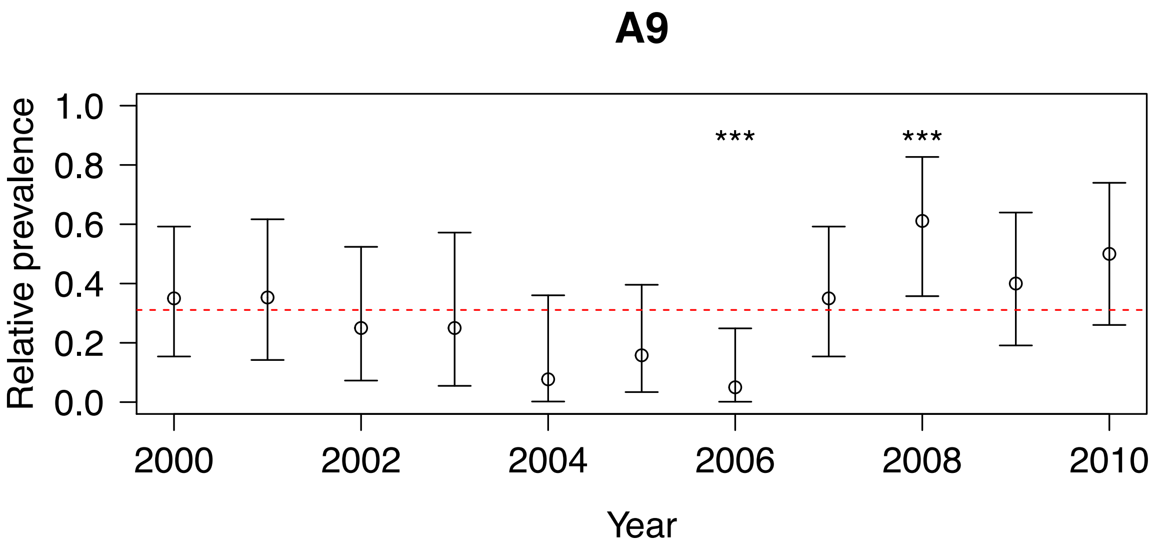

Figure S3b. The relative prevalences of *Borrelia afzelii* oMG A9 over the 11 years of the study. The relative prevalences were calculated as the proportion of nymphal tick-derived *B. afzelii* isolates infected with oMG A9. The bars indicate the 95% confidence interval and the red dotted line shows the mean relative prevalence of oMG A9 over the duration of the study. The asterisks indicate the years where the prevalences were significantly different ($\alpha = 0.05$) from the long-term mean.

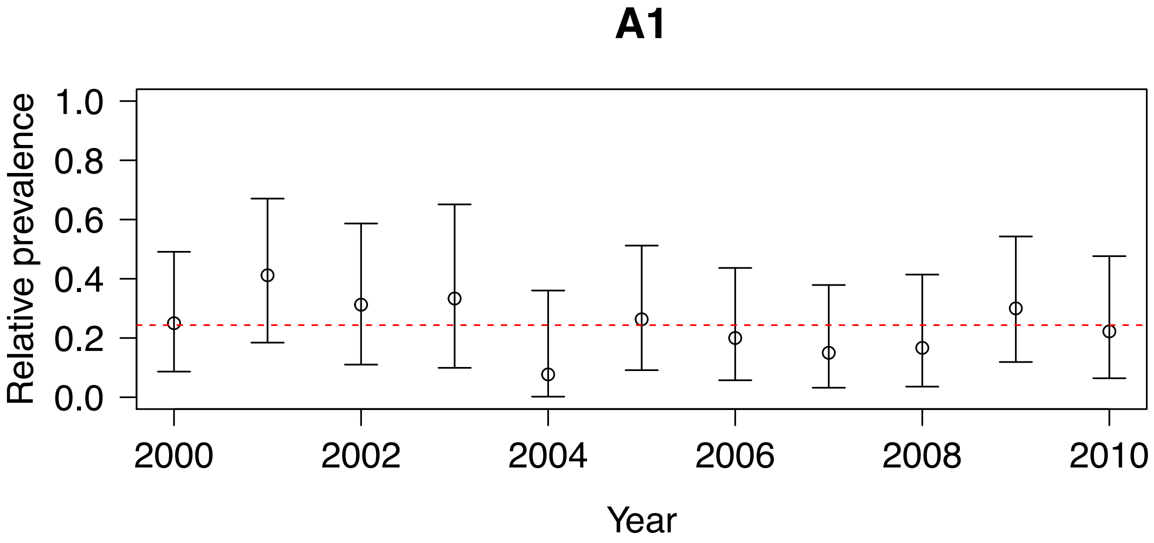

Figure S3c. The relative prevalences of *Borrelia afzelii* oMG A1 over the 11 years of the study. The relative prevalences were calculated as the proportion of nymphal tick-derived *B. afzelii* isolates infected with oMG A1. The bars indicate the 95% confidence interval and the red dotted line shows the mean relative prevalence of oMG A1 over the duration of the study. The asterisks indicate the years where the prevalences were significantly different ($\alpha = 0.05$) from the long-term mean.

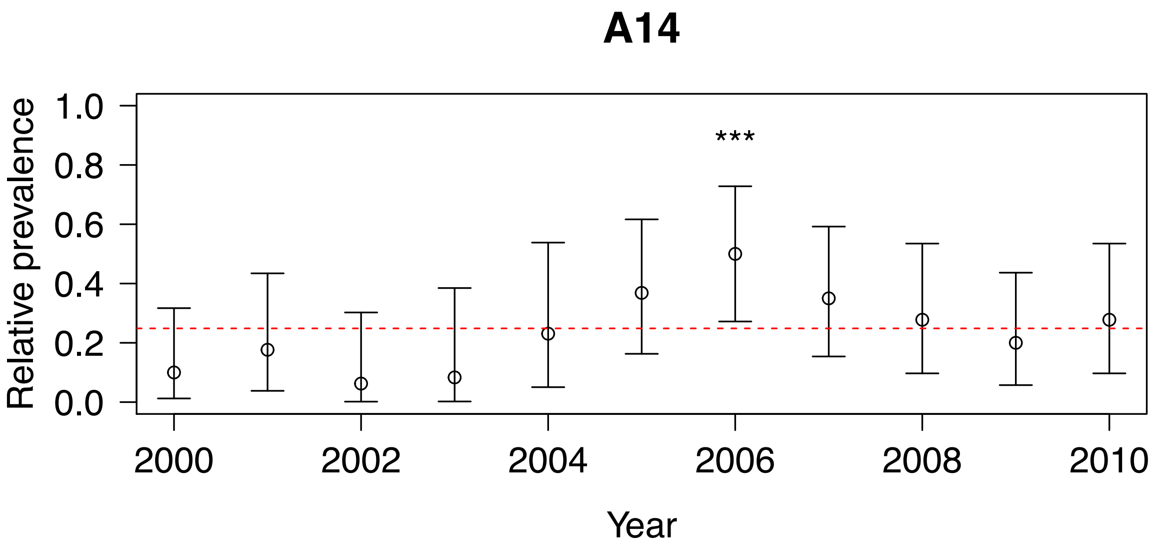

Figure S3d. The relative prevalences of *Borrelia afzelii* oMG A14 over the 11 years of the study. The relative prevalences were calculated as the proportion of nymphal tick-derived *B. afzelii* isolates infected with oMG A14. The bars indicate the 95% confidence interval and the red dotted line shows the mean relative prevalence of oMG A14 over the duration of the study. The asterisks indicate the years where the prevalences were significantly different ($\alpha = 0.05$) from the long-term mean.

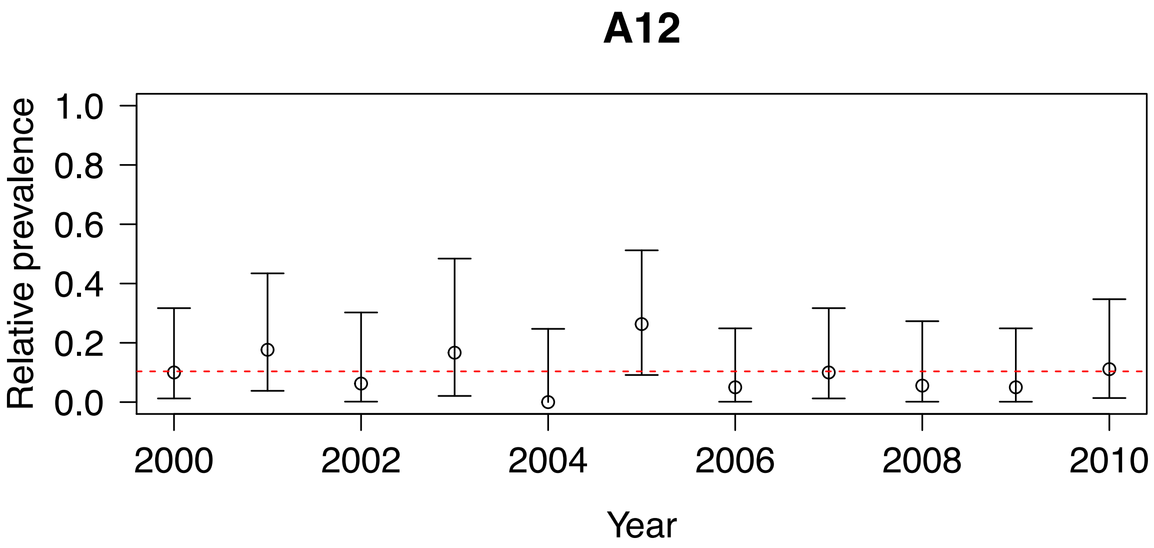

Figure S3e. The relative prevalences of *Borrelia afzelii* oMG A12 over the 11 years of the study. The relative prevalences were calculated as the proportion of nymphal tick-derived *B. afzelii* isolates infected with oMG A12. The bars indicate the 95% confidence interval and the red dotted line shows the mean relative prevalence of oMG A12 over the duration of the study. The asterisks indicate the years where the prevalences were significantly different (α = 0.05) from the long-term mean.

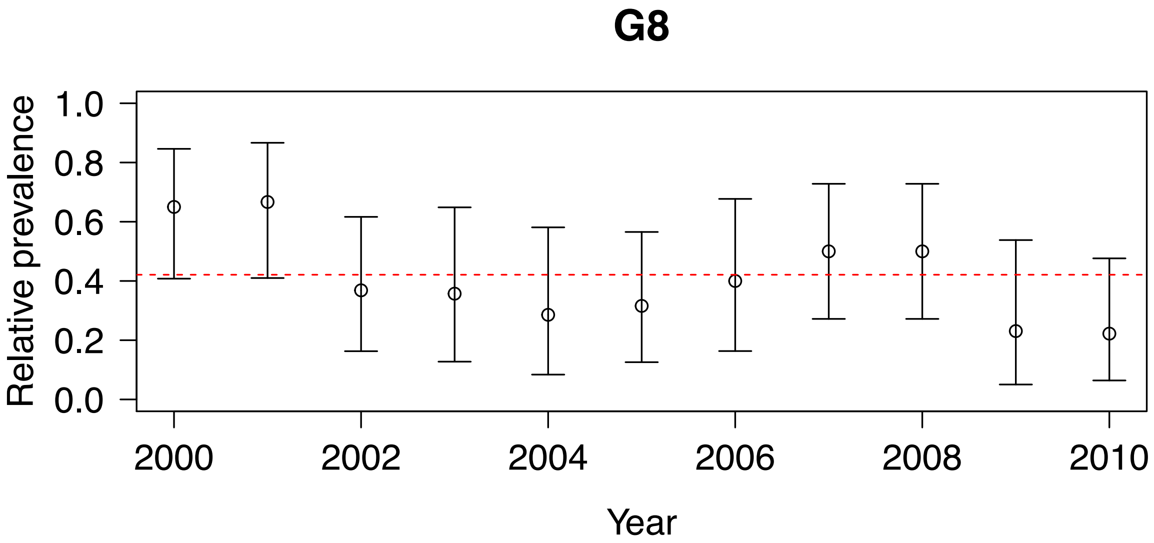

Figure S4a. The relative prevalences of *Borrelia garinii* oMG G8 over the 11 years of the study. The relative prevalences were calculated as the proportion of nymphal tick-derived *B. garinii* isolates infected with oMG G8. The bars indicate the 95% confidence interval and the red dotted line shows the mean relative prevalence of oMG G8 over the duration of the study. The asterisks indicate the years where the prevalences were significantly different ($\alpha = 0.05$) from the long-term mean.

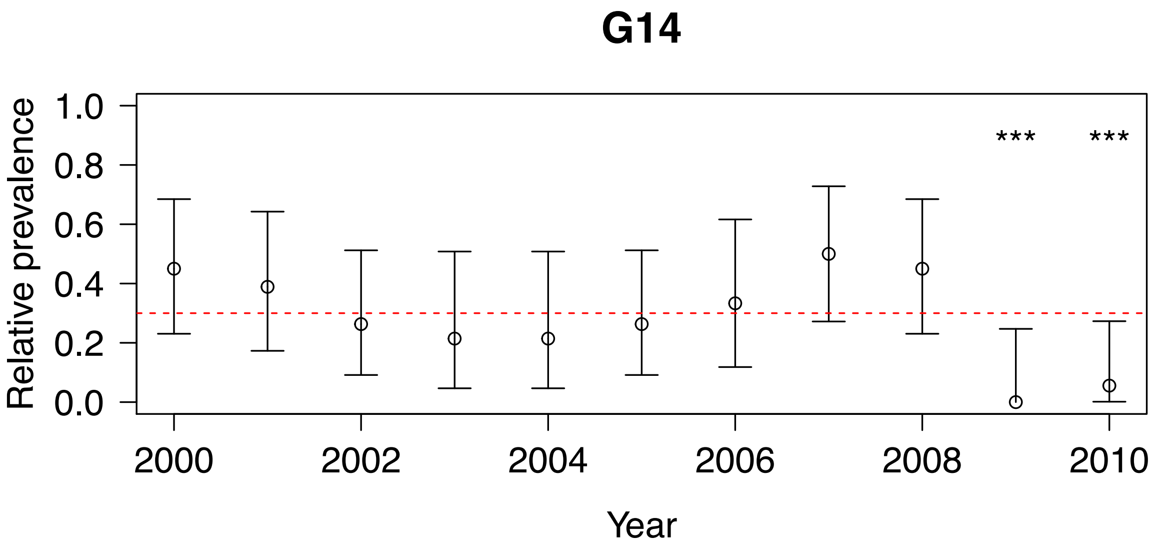

Figure S4b. The relative prevalences of *Borrelia garinii* oMG G14 over the 11 years of the study. The relative prevalences were calculated as the proportion of nymphal tick-derived *B. garinii* isolates infected with oMG G14. The bars indicate the 95% confidence interval and the red dotted line shows the mean relative prevalence of oMG G14 over the duration of the study. The asterisks indicate the years where the prevalences were significantly different (α = 0.05) from the long-term mean.

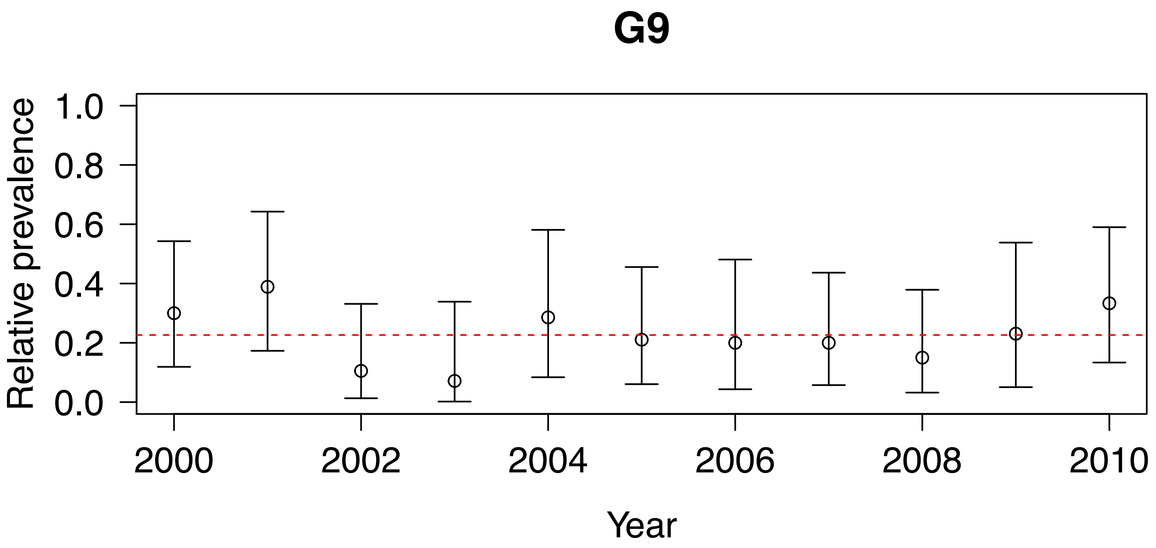

Figure S4c. The relative prevalences of *Borrelia garinii* oMG G9 over the 11 years of the study. The relative prevalences were calculated as the proportion of nymphal tick-derived *B. garinii* isolates infected with oMG G9. The bars indicate the 95% confidence interval and the red dotted line shows the mean relative prevalence of oMG G9 over the duration of the study. The asterisks indicate the years where the prevalences were significantly different ($\alpha = 0.05$) from the long-term mean.

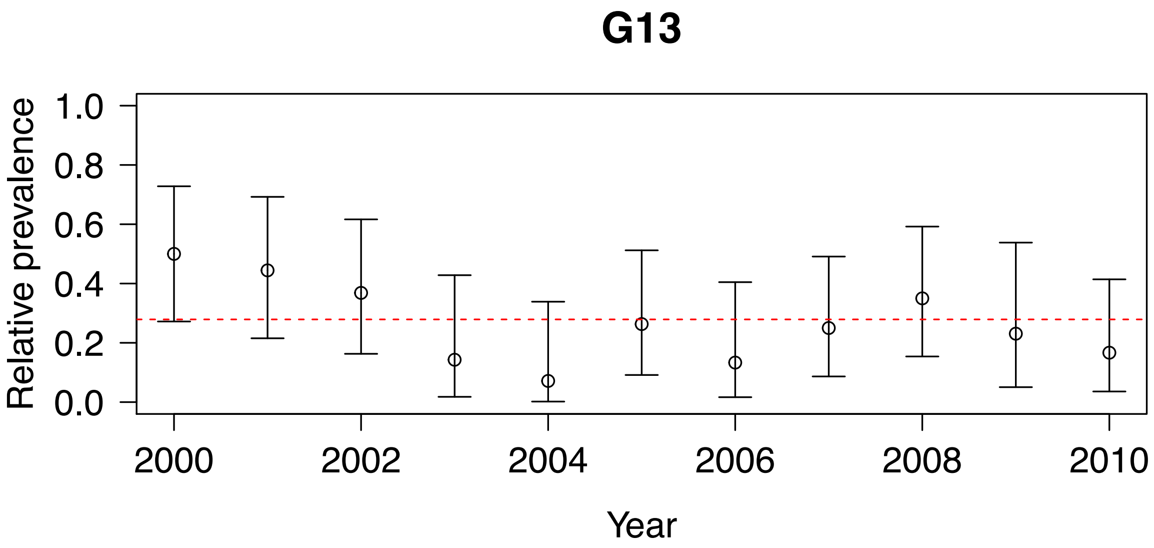

Figure S4d. The relative prevalences of *Borrelia garinii* oMG G13 over the 11 years of the study. The relative prevalences were calculated as the proportion of nymphal tick-derived *B. garinii* isolates infected with oMG G13. The bars indicate the 95% confidence interval and the red dotted line shows the mean relative prevalence of oMG G13 over the duration of the study. The asterisks indicate the years where the prevalences were significantly different (α = 0.05) from the long-term mean.

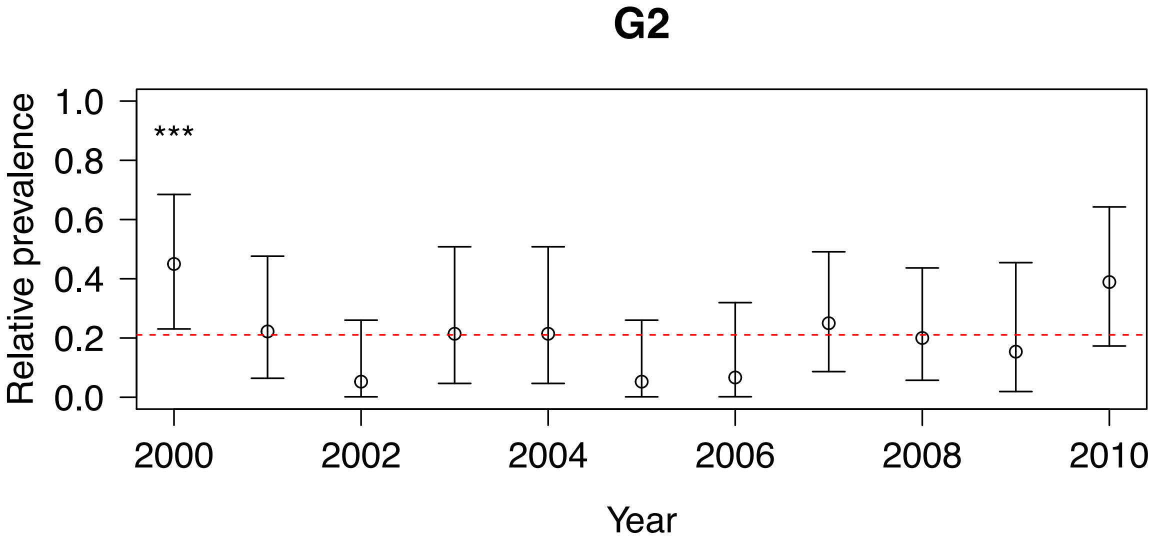

Figure S4e. The relative prevalences of *Borrelia garinii* oMG G2 over the 11 years of the study. The relative prevalences were calculated as the proportion of nymphal tick-derived *B. garinii* isolates infected with oMG G2. The bars indicate the 95% confidence interval and the red dotted line shows the mean relative prevalence of oMG G2 over the duration of the study. The asterisks indicate the years where the prevalences were significantly different ($\alpha = 0.05$) from the long-term mean.

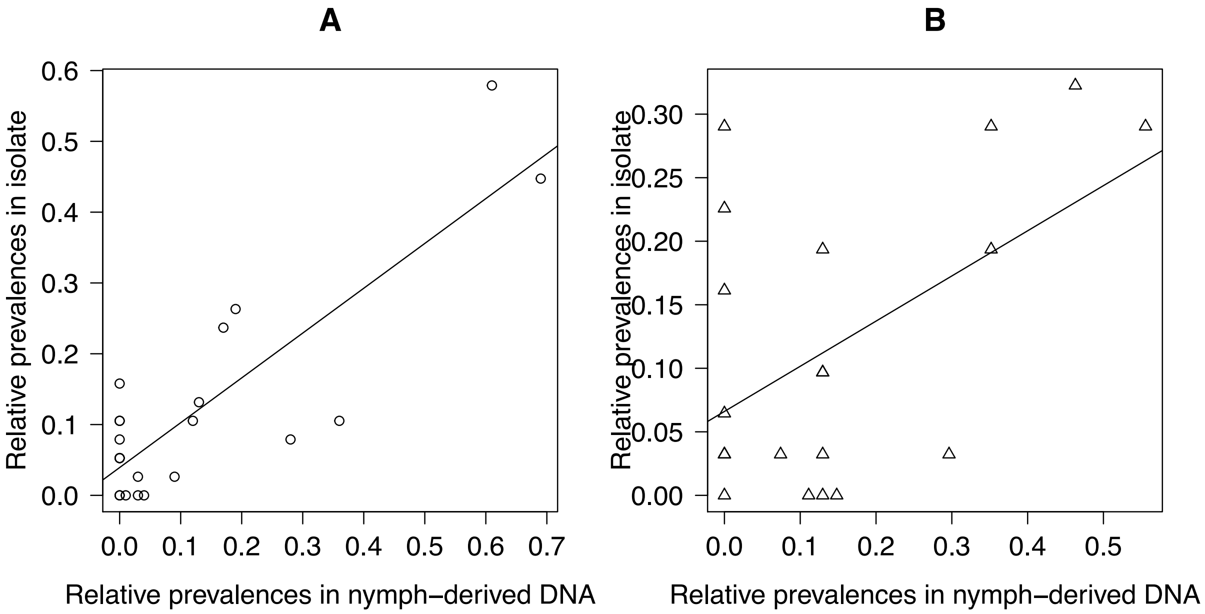

Figure S5. The origin of the *Borrelia* DNA extraction has no effect on the relative prevalences of the oMG strains. The relationship between the relative prevalences of the oMGs (combined for 2009 and 2010) using DNA from nymph-derived isolates cultured in BSK medium versus DNA directly extracted from the nymphs is shown for both (A) *B. afzelii* and (B) *B. garinii*. The Pearson correlation of the relative prevalence distribution of the oMG strains between the two DNA extraction methods was highly significant in both *B. afzelii* ($r = 0.845$, $t = 7.056$, $df = 20$, $p < 0.001$) and *B. garinii* ($r = 0.516$, $t = 2.559$, $df = 18$, $p = 0.019$).

References

1. Durand, J. *et al.* Cross-immunity and community structure of a multiple-strain pathogen in the tick vector. *Appl Environ Microbiol* **81**, 7740-7752, doi:10.1128/aem.02296-15 (2015).
2. Andersson, M., Scherman, K. & Raberg, L. Multiple-strain infections of *Borrelia afzelii*: a role for within-host interactions in the maintenance of antigenic diversity? *Am Nat* **181**, 545-554 (2013).
3. Hellgren, O., Andersson, M. & Raberg, L. The genetic structure of *Borrelia afzelii* varies with geographic but not ecological sampling scale. *J Evol Biol* **24**, 159-167 (2011).
4. Strandh, M. & Raberg, L. Within-host competition between *Borrelia afzelii ospC* strains in wild hosts as revealed by massively parallel amplicon sequencing. *Philos T Roy Soc B* **370**, doi:10.1098/rstb.2014.0293 (2015).
5. Durand, J. *et al.* Multi-strain infections of the Lyme borreliosis pathogen in the tick vector. *Appl Environ Microbiol* **83** (2016).
6. Bunikis, J. *et al.* Sequence typing reveals extensive strain diversity of the Lyme borreliosis agents *Borrelia burgdorferi* in North America and *Borrelia afzelii* in Europe. *Microbiology-Sgm* **150**, 1741-1755, doi:10.1099/mic.0.26944-0 (2004).
7. Dykhuizen, D. E. & Baranton, G. The implications of a low rate of horizontal transfer in *Borrelia*. *Trends Microbiol* **9**, 344-350, doi:10.1016/s0966-842x(01)02066-2 (2001).
8. Wang, G. Q., van Dam, A. P. & Dankert, J. Evidence for frequent OspC gene transfer between *Borrelia valaisiana* sp nov and other Lyme disease spirochetes. *FEMS Microbiol Lett* **177**, 289-296, doi:10.1016/s0378-1097(99)00346-8 (1999).
9. Qiu, W. G. *et al.* Genetic exchange and plasmid transfers in *Borrelia burgdorferi* sensu stricto revealed by three-way genome comparisons and multilocus sequence typing. *Proc Natl Acad Sci U S A* **101**, 14150-14155, doi:10.1073/pnas.0402745101 (2004).
10. Attie, O. *et al.* Co-evolution of the outer surface protein C gene (*ospC*) and intraspecific lineages of *Borrelia burgdorferi* sensu stricto in the northeastern United States. *Infection Genetics and Evolution* **7**, 1-12, doi:10.1016/j.meegid.2006.02.008 (2007).
11. Margos, G. *et al.* MLST of housekeeping genes captures geographic population structure and suggests a European origin of *Borrelia burgdorferi*. *Proc Natl Acad Sci U S A* **105**, 8730-8735, doi:10.1073/pnas.0800323105 (2008).
12. Herrmann, C., Gern, L. & Voordouw, M. Species co-occurrence patterns among Lyme borreliosis pathogens in the tick vector *Ixodes ricinus*. *Appl Environ Microbiol* **79**, 7273-7280 (2013).
13. Morán Cadenas, F. *et al.* A comparison of two DNA extraction approaches in the detection of *Borrelia burgdorferi* sensu lato from live *Ixodes ricinus* ticks by PCR and reverse line blotting. *Vector-Borne Zoonot* **7**, 555-561, doi:10.1089/vbz.2006.0596 (2007).
14. Tonetti, N., Voordouw, M. J., Durand, J., Monnier, S. & Gern, L. Genetic variation in transmission success of the Lyme borreliosis pathogen *Borrelia afzelii*. *Ticks and Tick-Borne Diseases* **6**, 334-343 (2015).
